# Trends in Government‐Subsidised Menopausal Hormone Therapy Dispensing in Australia Between 2014 and 2023

**DOI:** 10.1111/ajo.70135

**Published:** 2026-04-24

**Authors:** Hindun Wilda Risni, Li Wei, Martha Hickey, Luke Grzeskowiak, Jenni Ilomäki, Ruth Brauer

**Affiliations:** ^1^ Research Department of Practice and Policy University College London School of Pharmacy London UK; ^2^ The University of Melbourne Parkville Victoria Australia; ^3^ Menopause Services, the Gynaecology Research Centre at the Royal Women's Hospital Parkville Victoria Australia; ^4^ College of Medicine and Public Health, Flinders Health and Medical Research Institute, Flinders University Bedford Park South Australia Australia; ^5^ SAHMRI Women and Kids, South Australian Health and Medical Research Institute, Women's and Children's Hospital North Adelaide South Australia Australia; ^6^ Centre for Medicine Use and Safety, Monash University Melbourne Victoria Australia; ^7^ School of Public Health and Preventive Medicine, Faculty of Medicine, Nursing, and Health Sciences, Monash University Melbourne Victoria Australia

**Keywords:** Australia, dispensing, menopausal hormone therapy, trend, women

## Abstract

Menopausal hormone therapy (MHT) is a mainstay treatment for menopausal symptoms. While international studies report rising MHT use, trends in Australia remain unclear. Using a 10% random sample of Pharmaceutical Benefits Scheme (PBS) data from 2014 to 2023, we analysed the prevalence of MHT dispensing among women aged 45–64. Overall prevalence of MHT dispensing remained stable (relative annual change: 0.42%, 95% CI −0.50 to 1.35). Use of transdermal and intrauterine device (IUD) MHT increased (5.89%, 95% CI 3.88–7.91 and 10.22%, 95% CI 9.48–10.95, respectively), and vaginal MHT decreased (−1.47%, 95% CI −2.30 to −0.63), while oral MHT appeared stable (0.66%, 95% CI −0.04 to 1.36). This study offers a clearer understanding of how MHT use in Australia has changed over the last decade.

## 1 Introduction

Women experiencing menopause may face a range of symptoms that can impact their quality of life [1]. Multiple guidelines recommend menopausal hormone therapy (MHT) for symptom relief, with primary indications for MHT being vasomotor symptoms, genitourinary symptoms, mood disturbance and bone loss prevention [2]. The International Menopause Society highlights the need for personalised MHT use, considering symptoms, medical history and patient preferences [3]. MHT use declined sharply following the Women's Health Initiative trial, [4, 5] but recent international studies indicate a renewed upward trend. In the United Kingdom, MHT incidence has increased from 2010 to 2021 [6]. The English National Health Service (NHS) reported a 47% rise in MHT use in 2022–2023 compared with the previous year [7]. In Sweden, after a plateau in 2007–2016, MHT prescribing also increased from 2017 to 2021 [8]. However, it is unclear whether similar trends are reflected in Australia. This study aimed to describe changes in the prevalence of government‐subsidised MHT dispensing using the Pharmaceutical Benefits Scheme (PBS) database among Australian women aged 45–64.

## 2 Methods

The PBS is a Commonwealth scheme that subsidises prescription medicines for Australian residents and eligible foreign visitors [9]. For the current study, a 10% random sample of data from PBS was used. Our study cohort comprised women aged 45–64 who were dispensed MHT between 01 January 2014 and 31 December 2023. We identified MHT based on ATC and PBS item codes, including oestrogen, progestins and their combinations in any strength. We then stratified the analysis by age, route and type of administration. The routes of administration included the oral route (tablets and capsules), transdermal route (patches), vaginal route (vaginal tablet, cream, pessaries and gel) and intrauterine device (IUD). Types of administration included oestrogen‐only, progestin‐only and combination. We analysed the route and type of MHT based on the individual items. However, if oestrogen and progestin were dispensed separately within the same year, this was classified as combination therapy. Use of levonorgestrel alone was excluded from the analysis, as it is most likely prescribed for contraception rather than for treating menopausal symptoms.

Prevalent use was defined as at least one dispensing record per calendar year. We calculated prevalence using age‐specific mid‐year population estimates from the Australian Bureau of Statistics [10]. The annual prevalence was expressed as a percentage (per 100 individuals) and 95% confidence interval (CI), estimated by the Poisson method. Assuming an underlying linear relationship, relative average annual changes between 2014 and 2023 were assessed using a linear regression model and stratified by different age groups and routes of MHT. Analyses were conducted using Microsoft Excel and StataNow MP 18.5 (StataCorp LLC). As an additional analysis, we performed joinpoint regression analyses to identify changes in trend and their annual percent change using the Joinpoint Regression Program (version 5.4.0) developed by the United States. National Cancer Institute (NCI). This study was approved by the Monash University Human Research Ethics Committee (ID 22877).

## 3 Results and Discussion

**FIGURE 1:**
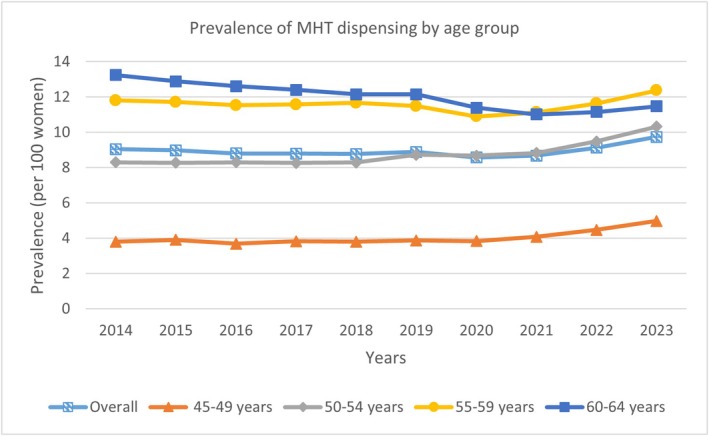
Prevalence of MHT dispensing by age group between 2014 and 2023.

The overall prevalence of PBS MHT dispensing showed a stable trend (Figure 1), from 9.05% (95% CI 8.94–9.15) in 2014 to 9.74% (95% CI 9.63–9.84) in 2023, a relative annual change of 0.42% (95% CI −0.50 to 1.35). When stratified by age, the highest relative annual increase was observed in women aged 45–49, from 3.80% (95% CI 3.67–3.94) to 4.98% (95% CI 4.83–5.12), a relative annual increase of 2.50% (95% CI 0.86–4.15). A gradual annual increase in MHT use was also observed in women aged 50–54 (relative annual increase 2.14%, 95% CI 1.06–3.21). Overall MHT use decreased in women aged 60–64 (relative annual decrease −1.92%, 95% CI −2.50 to −1.34). The decline of MHT use in women aged 60–64, although slight, may reflect heightened risk considerations associated with MHT in older age [11, 12, 13].

**FIGURE 2:**
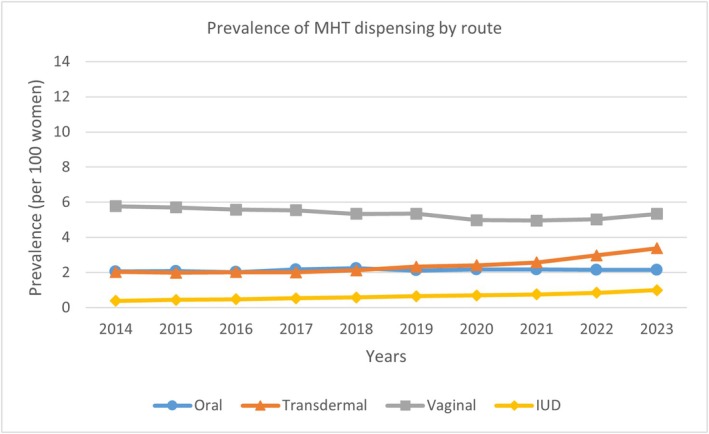
Prevalence of MHT dispensing by route between 2014 and 2023.

The dispensing of vaginal MHT slightly decreased from 2014 (5.77%, 95% CI 5.69–5.86) to 2023 (5.33%, 95% CI 5.25–5.41), a relative annual decrease of −1.47% (−2.30 to −0.63). The oral route showed a stable trend, from 2.05% (95% CI 2.00–2.10) in 2014 to 2.15% (95% CI 2.10–2.20) in 2023, a relative annual change of 0.66% (95% CI −0.04 to 1.36). The dispensing of transdermal MHT increased from 2.03% (95% CI 1.98–2.08) in 2014 to 3.38% (95% CI 3.32–3.44) in 2023, a relative annual increase of 5.89% (95% CI 3.88–7.91) (Figure 2). Although low in prevalence, the largest increasing trend was observed in IUD use, from 0.39% (0.37–0.41) in 2014 to 0.99% (0.96–1.02) in 2023, a relative annual increase of 10.22% (95% CI 9.48–10.95). When stratified by age, increased dispensing of transdermal and IUD MHT was measured in all age groups (see Supporting Information), with the highest relative increase in transdermal use among women aged 50–54: from 2.61% (95% CI 2.50–2.72) in 2014 to 4.96% (95% CI 4.82–5.11) in 2023 (7.67% [95% CI 5.27–10.08] relative increase). A similar increase was also seen for women aged 45–49: from 1.06% (95% CI 0.99–1.13) in 2014 to 2.03 (95% CI 1.93–2.13) in 2023 (7.24% [95% CI 5.16–9.32] relative increase).

Joinpoint analyses were performed to identify a change in trend. An increased trend in overall MHT use was found between 2021 and 2023, with an annual percentage change (APC) of 6.15 (95% CI 4.31–7.39). After a plateau from 2014 to 2018 (APC 0.73 [95% CI −5.85 to 4.09]), the transdermal route showed an upward trend between 2018 and 2023 (APC of 9.64 [95% CI 7.13–16.81]). IUD use also increased, with a higher APC observed between 2021 and 2023 (13.79 [95% CI 9.9–16.54]) compared with earlier years (see Supporting Information). The oral route exhibited a stable trend, while the dispensing of vaginal MHT decreased (see Supporting Information).

The rising use of transdermal MHT across all ages may be partly driven by evidence of its greater safety compared with oral MHT, especially for venous thromboembolic risk (VTE) [14]. IMS 2016 recommends against oral oestrogen in women with a personal history of VTE and recommends transdermal oestrogen as the first choice in obese women with menopausal symptoms [3]. Other clinical guidelines, such as those from the National Institute for Health and Care Excellence (NICE), United Kingdom, and the Endocrine Society, United States of America, mention stroke and VTE as possible adverse effects of MHT but also note that these risks are lower with transdermal oestrogen [2]. Moreover, an increase in IUD use was seen. In recent years, there have been some discussions regarding the use of LNG‐IUD as part of MHT [15, 16, 17]. When combined with an oestrogen, the LNG‐IUD may offer an effective approach to managing both contraceptive needs and vasomotor symptoms during the perimenopausal transition [15]. LNG‐IUD can be used up to five years to protect the endometrium in women who use oestrogen for menopausal symptoms [17].

Systemic MHT is primarily used for vasomotor symptoms, while local MHT, such as vaginal oestrogen, is often prescribed to alleviate urogenital symptoms. The use of local oestrogen minimises the systemic absorption and therefore does not need the additional use of progesterone when an appropriate low dose is used [3]. In this study, oestrogen‐only preparations were the most frequently dispensed formulation (see Supplement), likely reflecting the high use of vaginal oestrogen among older postmenopausal women in whom urogenital symptoms are common [18].

The observed stable trend in overall MHT prevalence alongside an increasing trend in the use of transdermal and IUD MHT suggests that women are utilising MHT to manage menopausal symptoms. While the risks and benefits of MHT are known to be influenced by individual co‐morbidities, formulation, dosage and duration of use, [19] a qualitative study in 2020 involving 32 Australian women revealed a reluctance towards MHT use [20]. Despite this hesitancy, women expressed a willingness to receive reliable information about menopause [20]. Therefore, ensuring the consistent provision of evidence‐based information to women in Australia regarding menopause and the associated risks and benefits of MHT remains crucial.

Our findings offer timely insights into MHT dispensing trends, particularly in the context of recent initiatives in Australia. From March 2025, newer MHT products have been added to the PBS, enhancing affordability and availability for Australian women. This study provides a valuable baseline for future research evaluating the impact of expanded PBS coverage. Additionally, given Australia's ethnic diversity, future research should also explore MHT use across different ethnic groups.

## 4 Limitations

This study is the first to report MHT dispensing trends in Australia from 2014 to 2023. However, the lack of data on menopausal status and MHT indications may lead to overlapping therapeutic uses, potentially overestimating results. Additionally, the PBS database includes only government‐subsidised MHT formulations; thus, prescriptions outside PBS coverage were not captured. This is likely to have led to an underestimation of the true utilisation of MHT in these analyses. Moreover, there has been a national shortage of transdermal oestrogen since 2020. While certain products were only listed on the PBS from 2025, many clinicians were prescribing these products during the supply shortage.

It is important to note that this study provides descriptive data on MHT dispensing rates. Information on clinical factors, such as whether women had an intact uterus, was unavailable; therefore, we did not intend to assess the appropriateness of dispensed MHT.

## Funding

HWR received a scholarship from the Indonesia Endowment Fund for Education (LPDP No. 202307223267940), Ministry of Finance, Republic of Indonesia, for her study. RB received a Pharmalliance ECR to support a work visit at Monash University.

## Conflicts of Interest

The authors declare no conflicts of interest.

## Supporting information


**Table S1:** Annual prevalence of oral MHT by age.
**Table S2:** Annual prevalence of transdermal MHT by age.
**Table S3:** Annual prevalence of vaginal MHT by age.
**Table S4:** Annual prevalence of IUD MHT by age.
**Table S5:** List of PBS‐subsidised MHT.
**Figure S1:** Prevalence of MHT dispensing by type.
**Figure S2:** Trend of overall MHT use.
**Figure S3:** Trend of oral MHT use.
**Figure S4:** Trend of transdermal MHT use.
**Figure S5:** Trend of vaginal MHT use.
**Figure S6:** Trend of IUD MHT use.

## Data Availability

The data that support the findings of this study are available from the corresponding author upon reasonable request.
