# Supplementary material for: Trends in Government‐Subsidised Menopausal Hormone Therapy Dispensing in Australia Between 2014 and 2023
Source: Aust N Z J Obstet Gynaecol. 2026 Apr 24;66:e70135. doi: 10.1111/ajo.70135 (PMC13109606; doi:10.1111/ajo.70135)
Supplement: Supplementary file 1 — Table S1: Annual prevalence of oral MHT by age. Table S2: Annual prevalence of transdermal MHT by age. Table S3: Annual prevalence of vaginal MHT by age. Table S4: Annual prevalence of IUD MHT by age. Table S5: List of PBS‐subsidised MHT. Figure S1: Prevalence of MHT dispensing by type. Figure S2: Trend of overall MHT use. Figure S3: Trend of oral MHT use. Figure S4: Trend of transdermal MHT use. Figure S5: Trend of vaginal MHT use. Figure S6: Trend of IUD MHT use. [file AJO-66-0-s001.docx]

**Supplementary materials**

**Table 1. Annual prevalence of oral MHT by age**

| **Year** | **Prevalence % (95% CI)** | | | | |
| --- | --- | --- | --- | --- | --- |
|  | **overall** | **45-49** | **50-54** | **55-59** | **60-64** |
| 2014 | 2.05 (2.00-2.10) | 2.30 (2.20-2.41) | 2.64 (2.52-2.75) | 1.81 (1.72-1.91) | 1.28 (1.19-1.37) |
| 2015 | 2.08 (2.03-2.13) | 2.44 (2.33-2.55) | 2.68 (2.56-2.79) | 1.83 (1.73-1.92) | 1.19 (1.11-1.27) |
| 2016 | 2.02 (1.97-2.07) | 2.22 (2.12-2.32) | 2.70 (2.58-2.81) | 1.86 (1.77-1.96) | 1.16 (1.08-1.25) |
| 2017 | 2.17 (2.12-2.22) | 2.34 (2.23-2.44) | 2.88 (2.76-3.00) | 2.08 (1.98-2.18) | 1.25 (1.17-1.34) |
| 2018 | 2.24 (2.19-2.29) | 2.33 (2.23-2.43) | 2.92 (2.80-3.04) | 2.24 (2.13-2.34) | 1.37 (1.29-1.46) |
| 2019 | 2.11 (2.06-2.16) | 2.17 (2.07-2.26) | 2.94 (2.82-3.06) | 2.00 (1.90-2.09) | 1.25 (1.17-1.33) |
| 2020 | 2.17 (2.12-2.22) | 2.14 (2.04-2.23) | 3.01 (2.89-3.13) | 2.19 (2.09-2.29) | 1.28 (1.20-1.36) |
| 2021 | 2.17 (2.12-2.22) | 2.22 (2.12-2.32) | 2.91 (2.80-3.03) | 2.24 (2.13-2.34) | 1.25 (1.17-1.33) |
| 2022 | 2.15 (2.10-2.20) | 2.32 (2.22-2.42) | 2.90 (2.79-3.01) | 2.15 (2.04-2.25) | 1.16 (1.08-1.23) |
| 2023 | 2.15 (2.10-2.20) | 2.29 (2.19-2.40) | 2.92 (2.80-3.03) | 2.20 (2.10-2.30) | 1.09 (1.02-1.16) |
| Relative annual change  (95% CI) | 0.66% (-0.04-1.36) | -0.31% (-1.29-0.66) | 1.24% (0.49-1.98) | 2.63% (1.31-3.95) | -0.55% (-2.20-1.10) |

**Table 2. Annual prevalence of transdermal MHT by age**

| **Year** | **Prevalence % (95% CI)** | | | | |
| --- | --- | --- | --- | --- | --- |
|  | **overall** | **45-49** | **50-54** | **55-59** | **60-64** |
| 2014 | 2.03 (1.98-2.08) | 1.06 (0.99-1.13) | 2.61 (2.50-2.72) | 2.51 (2.40-2.63) | 1.95 (1.84-2.06) |
| 2015 | 1.99 (1.94-2.04) | 1.05 (0.97-1.12) | 2.56 (2.45-2.67) | 2.58 (2.46-2.69) | 1.79 (1.68-1.89) |
| 2016 | 2.02 (1.97-2.07) | 1.09 (1.02-1.17) | 2.66 (2.54-2.77) | 2.67 (2.56-2.79) | 1.67 (1.57-1.76) |
| 2017 | 2.01 (1.96-2.06) | 1.15 (1.08-1.22) | 2.59 (2.48-2.71) | 2.66 (2.55-2.78) | 1.65 (1.55-1.74) |
| 2018 | 2.12 (2.07-2.17) | 1.19 (1.12-1.27) | 2.75 (2.63-2.86) | 2.87 (2.75-2.98) | 1.73 (1.63-1.83) |
| 2019 | 2.33 (2.27-2.38) | 1.32 (1.24-1.39) | 3.18 (3.05-3.30) | 2.98 (2.87-3.10) | 1.87 (1.77-1.97) |
| 2020 | 2.41 (2.36-2.47) | 1.33 (1.25-1.41) | 3.36 (3.24-3.49) | 3.12 (3.00-3.24) | 1.87 (1.78-1.97) |
| 2021 | 2.57 (2.52-2.63) | 1.46 (1.38-1.54) | 3.62 (3.49-3.74) | 3.33 (3.21-3.46) | 1.86 (1.77-1.96) |
| 2022 | 2.98 (2.92-3.04) | 1.72 (1.63-1.81) | 4.30 (4.17-4.44) | 3.84 (3.70-3.97) | 2.00 (1.90-2.10) |
| 2023 | 3.38 (3.32-3.44) | 2.03 (1.93-2.13) | 4.96 (4.82-5.11) | 4.32 (4.18-4.47) | 2.12 (2.02-2.22) |
| Relative annual change  (95% CI) | 5.89% (3.88-7.91) | 7.24% (5.16-9.32) | 7.67% (5.27-10.08) | 5.88% (4.28-7.48) | 1.67% (-0.06-3.39) |

**Table 3. Annual prevalence of vaginal MHT by age**

| **Vaginal** | | | | | |
| --- | --- | --- | --- | --- | --- |
|  | **overall** | **45-49** | **50-54** | **55-59** | **60-64** |
| 2014 | 5.77 (5.69-5.86) | 1.17 (1.09-1.25) | 4.06 (3.92-4.20) | 8.28 (8.08-8.48) | 10.67 (10.43-10.90) |
| 2015 | 5.70 (5.61-5.78) | 1.16 (1.09-1.24) | 4.05 (3.91-4.19) | 8.12 (7.92-8.32) | 10.48 (10.24-10.71) |
| 2016 | 5.58 (5.49-5.66) | 1.15 (1.08-1.23) | 3.99 (3.86-4.13) | 7.84 (7.64-8.03) | 10.33 (10.10-10.56) |
| 2017 | 5.55 (5.46-5.63) | 1.17 (1.10-1.25) | 3.94 (3.81-4.08) | 7.86 (7.67-8.05) | 10.15 (9.931-0.38) |
| 2018 | 5.34 (5.26-5.42) | 1.06 (0.99-1.13) | 3.83 (3.69-3.96) | 7.64 (7.45-7.82) | 9.67 (9.45-9.89) |
| 2019 | 5.34 (5.26-5.42) | 1.19 (1.12-1.27) | 3.78 (3.65-3.92) | 7.45 (7.26-7.63) | 9.65 (9.44-9.87) |
| 2020 | 4.98 (4.90-5.05) | 1.17 (1.10-1.24) | 3.64 (3.51-3.77) | 6.71 (6.54-6.88) | 8.91 (8.71-9.12) |
| 2021 | 4.96 (4.88-5.03) | 1.25 (1.17-1.33) | 3.66 (3.54-3.79) | 6.72 (6.55-6.89) | 8.61 (8.41-8.81) |
| 2022 | 5.02 (4.95-5.10) | 1.30 (1.22-1.38) | 3.73 (3.60-3.86) | 6.78 (6.60-6.96) | 8.63 (8.43-8.82) |
| 2023 | 5.33 (5.25-5.41) | 1.54 (1.46-1.63) | 4.03 (3.90-4.16) | 7.14 (6.96-7.32) | 8.94 (8.74-9.14) |
| Relative annual change (95% CI) | -1.47%  (-2.30 to -0.63) | 2.60%  (0.59-4.61) | -0.76%  (-1.72-0.19) | -2.24%  (-3.19 to -1.29) | -2.50%  (-3.20 to -1.80) |

**Table 4. Annual prevalence of IUD MHT by age**

| **IUD** | | | | | |
| --- | --- | --- | --- | --- | --- |
|  | **overall** | **45-49** | **50-54** | **55-59** | **60-64** |
| 2014 | 0.39 (0.37-0.41) | 0.35 (0.31-0.39) | 0.67 (0.61-0.72) | 0.39 (0.34-0.43) | 0.10 (0.08-0.13) |
| 2015 | 0.45 (0.42-0.47) | 0.42 (0.38-0.47) | 0.74 (0.68-0.79) | 0.45 (0.40-0.49) | 0.13 (0.10-0.16) |
| 2016 | 0.47 (0.45-0.50) | 0.42 (0.38-0.47) | 0.76 (0.70-0.82) | 0.52 (0.47-0.57) | 0.14 (0.11-0.17) |
| 2017 | 0.53 (0.51-0.56) | 0.49 (0.44-0.53) | 0.86 (0.80-0.93) | 0.59 (0.53-0.64) | 0.16 (0.13-0.19) |
| 2018 | 0.58 (0.55-0.61) | 0.47 (0.42-0.51) | 0.99 (0.92-1.06) | 0.65 (0.60-0.71) | 0.18 (0.15-0.21) |
| 2019 | 0.64 (0.62-0.67) | 0.50 (0.46-0.55) | 1.14 (1.07-1.22) | 0.69 (0.64-0.75) | 0.21 (0.17-0.24) |
| 2020 | 0.69 (0.66-0.72) | 0.57 (0.52-0.62) | 1.22 (1.14-1.29) | 0.71 (0.65-0.77) | 0.25 (0.21-0.28) |
| 2021 | 0.75 (0.72-0.78) | 0.65 (0.59-0.70) | 1.26 (1.18-1.34) | 0.80 (0.73-0.86) | 0.27 (0.23-0.30) |
| 2022 | 0.84 (0.81-0.88) | 0.72 (0.66-0.78) | 1.43 (1.35-1.51) | 0.88 (0.81-0.95) | 0.30 (0.26-0.34) |
| 2023 | 0.99 (0.96-1.02) | 0.85 (0.78-0.91) | 1.66 (1.58-1.75) | 1.07 (0.99-1.14) | 0.32 (0.28-0.36) |
| Relative annual change (95% CI) | 10.22 (9.48-10.95) | 9.33 (7.56-11.11) | 10.67 (9.65-11.69) | 10.70 (9.25-12.16) | 13.54 (12.36-14.72) |

**Figure 1. Prevalence of MHT dispensing by type**

**JOINPOINT GRAPHS**


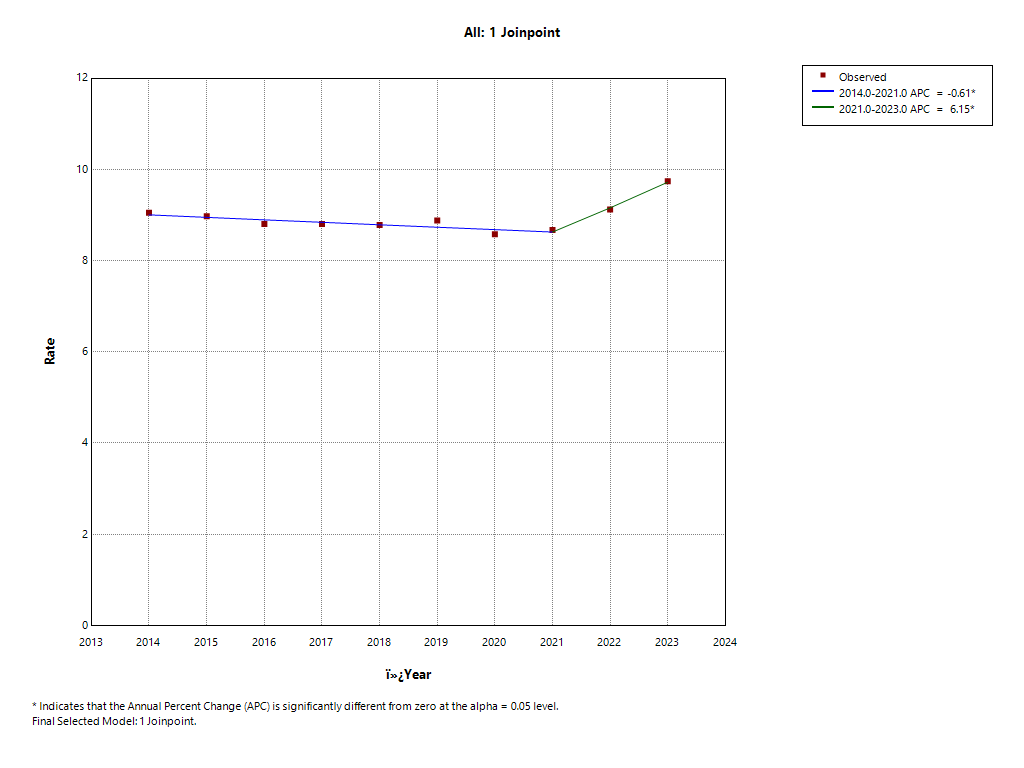


**Figure 2. Trend of overall MHT use**


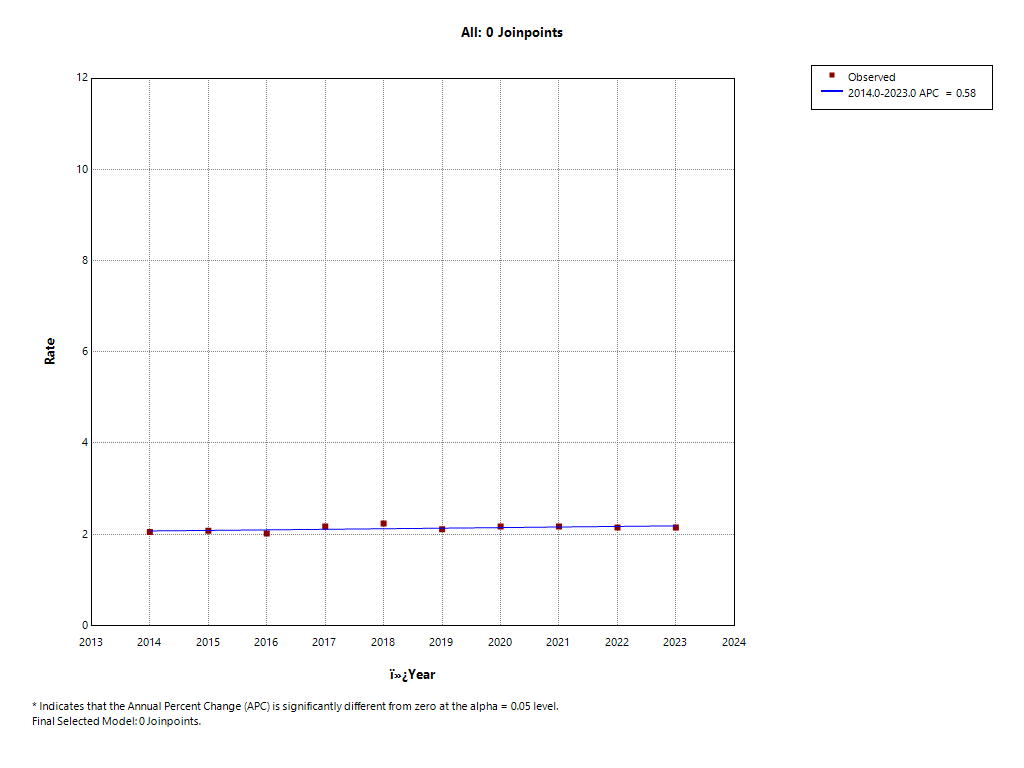


**Figure 3. Trend of oral MHT use**


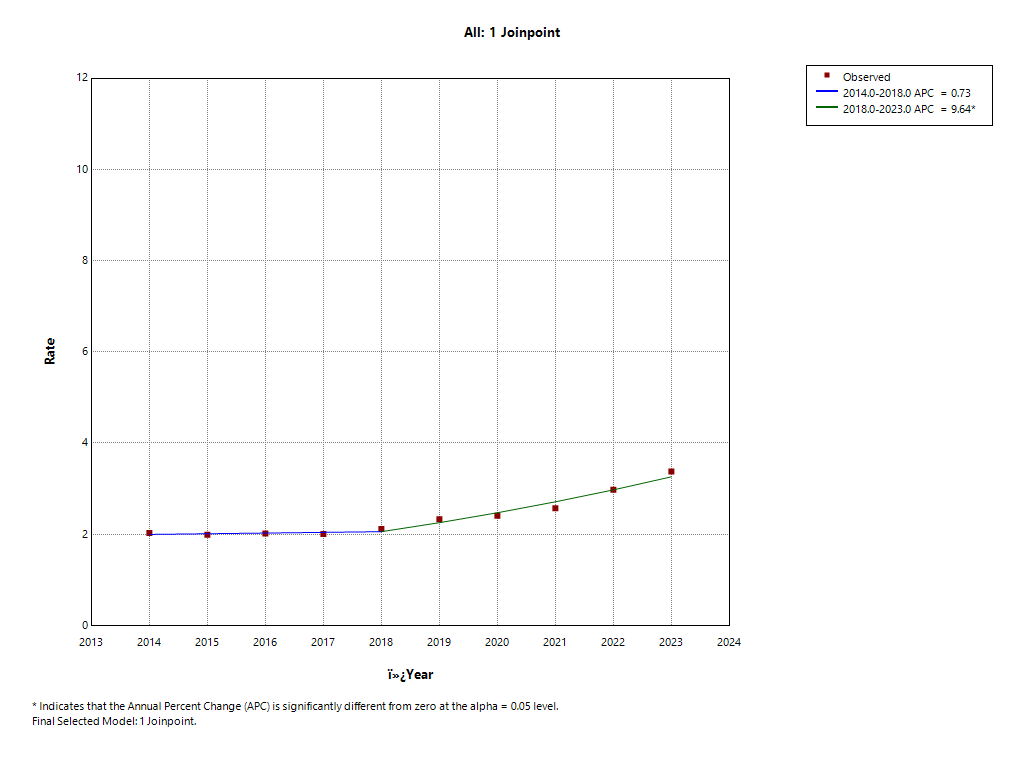


**Figure 4. Trend of transdermal MHT use**


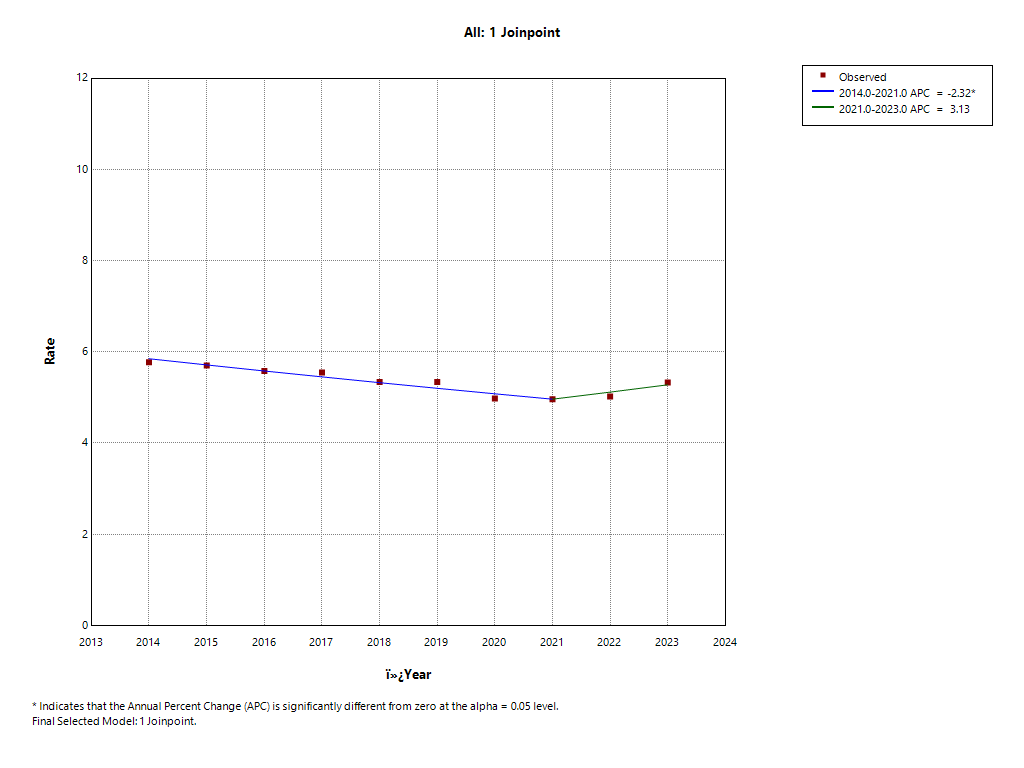


**Figure 5. Trend of vaginal MHT use**


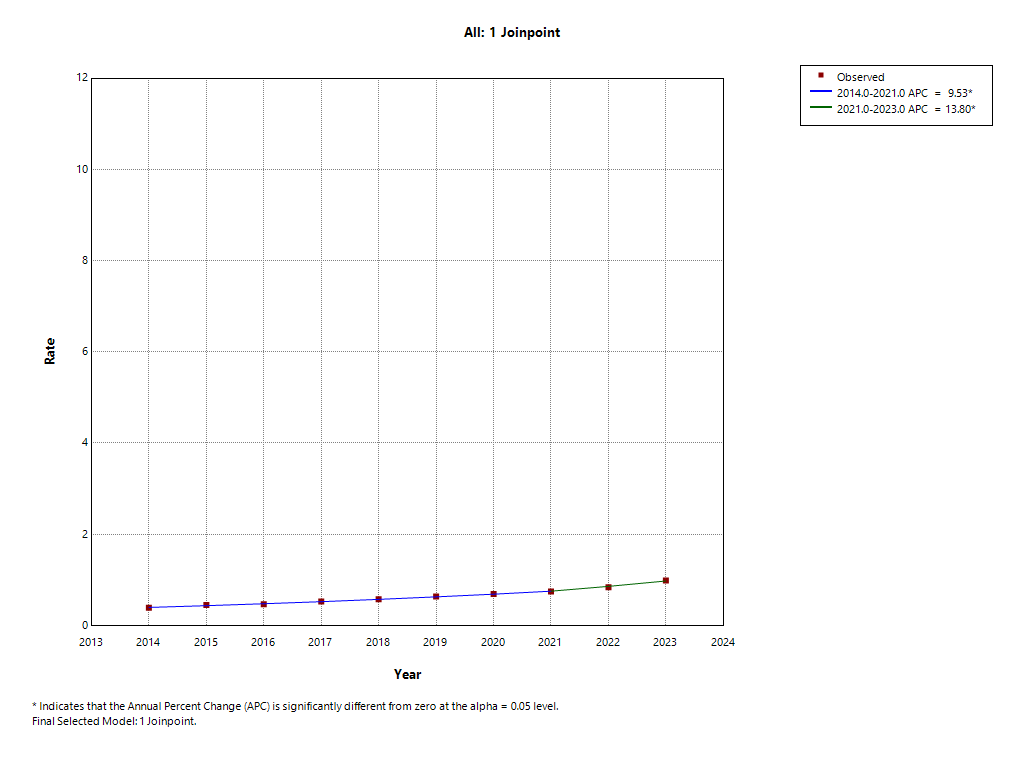


**Figure 6. Trend of IUD MHT use**

**Table 5. List of PBS subsidised MHT**

| **Item codes** | **Substance** | **Strength** |
| --- | --- | --- |
| **Oral** | | |
| 1664N | oestradiol | tablet 2 mg |
| 8274L | oestradiol | tablet 2 mg |
| 2993M | norethisterone | tablet 5 mg |
| 2722G | medroxyprogesterone | tablet 10 mg |
| 2321E | medroxyprogesterone | tablet 10 mg |
| 2323G | medroxyprogesterone | tablet 5 mg |
| 12598C | progesterone | capsule 200 mg |
| 10930G | progesterone | capsule 200 mg |
| 8244X | oestradiol + dydrogesterone | oestradiol 2 mg tablet + dydrogesterone 10 mg tablet |
| 10146B | oestradiol + dydrogesterone | oestradiol 1 mg tablet + dydrogesterone 10 mg tablet |
| 10142T | oestradiol + dydrogesterone | oestradiol 1 mg tablet + dydrogesterone 5 mg tablet |
| **Vaginal** |  |  |
| 1742Q | oestradiol | vaginal tablet 25 microgram |
| 10116K | progesterone | vaginal tablet 100 mg |
| 1781R | oestriol | vaginal cream 1 mg per g, 15 g |
| 6366C | progesterone | vaginal gel 90 mg |
| 1771F | oestriol | pessary 500 micrograms |
| 10203B | oestradiol | pessary 10 micrograms (as hemihydrate) |
| 12465C | progesterone | pessary 200 mg |
| 9608Q | progesterone | pessary 100 mg |
| 9609R | progesterone | pessary 200 mg |
| **Transdermal** | | |
| 8126Q | oestradiol | patches 7.6 mg |
| 8125P | oestradiol | patches 3.8 mg |
| 8140K | oestradiol | patches 1.5 mg (as hemihydrate) |
| 8286D | oestradiol | gel 1 mg (as hemihydrate) in 1 g sachet |
| 8311K | oestradiol | patches 750 micrograms (as hemihydrate) |
| 8312L | oestradiol | patches 3 mg (as hemihydrate) |
| 8425K | norethisterone acetate + oestradiol | patches 780 micrograms oestradiol (as hemihydrate), patches 620 micrograms estradiol (as hemihydrate), 2.7 mg norethisterone acetate |
| 8426L | norethisterone acetate + oestradiol | patches 780 micrograms oestradiol (as hemihydrate), patches 510 micrograms oestradiol (as hemihydrate), 4.8 mg norethisterone acetate |
| 8427M | oestradiol + norethisterone acetate | patches 620 micrograms oestradiol (as hemihydrate) with 2.7 mg norethisterone acetate |
| 8428N | oestradiol + norethisterone acetate | patches 510 micrograms oestradiol (as hemihydrate) with 4.8 mg norethisterone acetate |
| 8485N | oestradiol | patches 2 mg |
| 8486P | oestradiol | patches 5.7 mg |
| 8761D | oestradiol | patches 390 micrograms |
| 8762E | oestradiol | patches 585 micrograms |
| 8763F | oestradiol | patches 780 micrograms |
| 8764G | oestradiol | patches 1.17 mg |
| 8765H | oestradiol | patches 1.56 mg |
| **IUD (Intrauterine Device)** | | |
| 11909T | levonorgestrel | intrauterine drug delivery system 19.5 mg |
| 08633J | levonorgestrel | intrauterine drug delivery system 52 mg |
